# Flying Into the Wind: Insects and Bio-Inspired Micro-Air-Vehicles With a Wing-Stroke Dihedral Steer Passively Into Wind-Gusts

**DOI:** 10.3389/frobt.2022.820363

**Published:** 2022-02-24

**Authors:** Diana A. Olejnik, Florian T. Muijres, Matěj Karásek, Leonardo Honfi Camilo, Christophe De Wagter, Guido C.H.E. de Croon

**Affiliations:** ^1^ MAVLab, Department of Control and Operations, Delft University of Technology, Delft, Netherlands; ^2^ Experimental Zoology Group, Department of Animal Sciences, Wageningen University & Research, Wageningen, Netherlands

**Keywords:** fruit fly (*Drosophila*), MAV (micro air vehicle), flapping wing, CFD, aerodynamic, coupling, wingbeat, asymmetries

## Abstract

Natural fliers utilize passive and active flight control strategies to cope with windy conditions. This capability makes them incredibly agile and resistant to wind gusts. Here, we study how insects achieve this, by combining Computational Fluid Dynamics (CFD) analyses of flying fruit flies with freely-flying robotic experiments. The CFD analysis shows that flying flies are partly passively stable in side-wind conditions due to their dorsal-ventral wing-beat asymmetry defined as wing-stroke dihedral. Our robotic experiments confirm that this mechanism also stabilizes free-moving flapping robots with similar asymmetric dihedral wing-beats. This shows that both animals and robots with asymmetric wing-beats are dynamically stable in sideways wind gusts. Based on these results, we developed an improved model for the aerodynamic yaw and roll torques caused by the coupling between lateral motion and the stroke dihedral. The yaw coupling passively steers an asymmetric flapping flyer into the direction of a sideways wind gust; in contrast, roll torques are only stabilizing at high air gust velocities, due to non-linear coupling effects. The combined CFD simulations, robot experiments, and stability modeling help explain why the majority of flying insects exhibit wing-beats with positive stroke dihedral and can be used to develop more stable and robust flapping-wing Micro-Air-Vehicles.

## 1 Introduction

Tiny flying insects can operate in an enormous range of environmental conditions. They can fly in pitch darkness (Warrant, 2008), at high altitudes (Hu et al., 2016), travel surprisingly large distances (Chapman et al., 2015), they can navigate densely cluttered environments (Crall et al., 2015), and arguably most impressive, they can fly in highly-unsteady wind conditions (Combes and Dudley, 2009; Engels et al., 2016). These wind gusts can be larger than the maximum flight speed of the insect, and because of the small inertia of the insect, wind gusts can cause large linear and angular accelerations of the animal (Engels et al., 2019). Despite the enormous detrimental effects of winds on their flight control, many insects continue to fly in windy conditions, to collect food (Crall et al., 2017) or migrate (Leitch et al., 2021).

To be able to achieve this, the flight control system of insects relies on both passive and active flight stabilization mechanisms. The most important passive stabilization mechanisms are the aerodynamic flapping-wing counter-forces and counter-torques (Hedrick et al., 2009). But because these are insufficient and the flapping flight of insects is inherently unstable (Sun and Wang, 2007), these passive stabilization mechanisms need to be augmented with active neural-control-based stabilization. Due to the low inertia of insects, this neural flight control system needs to be both very precise and fast (Dickinson and Muijres, 2016). As a result, flying insects have sophisticated sensory systems (Taylor and Krapp, 2007), dedicated neural processing systems for flight (Christensen, 2004; Dickson et al., 2006; Fox et al., 2010), and sophisticated flapping-wing-based motor systems (Walker et al., 2014; Pratt et al., 2017). In addition to these active and passive mechanisms, insects can also make use of passive translation-induced aerodynamic torque coupling to control their rapid escape maneuvers (Karásek et al., 2018). This aerodynamic torque coupling is inherent to the nature of flapping wing-based propulsion. Because it is a passive byproduct of active control actions, this control system can be seen as a hybrid passive-active control system. The main research question of this study is whether insects flying in windy conditions can make use of a passive stabilization mechanisms similar to the one described in Karásek et al. (2018).

**FIGURE 1:**
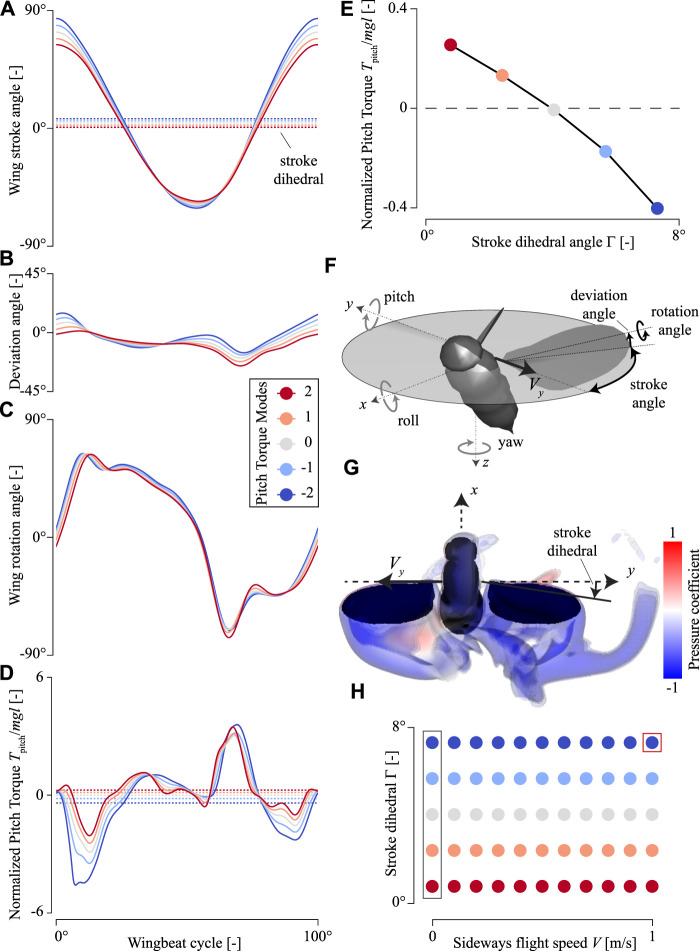
Temporal dynamics of the wing-beat kinematics and pitch torques produced by an in-silica hovering fruit fly, with variable pitch torque-producing wing-beat kinematics (Pitch Torque Modes). **(A–D)** Wing stroke angle, wing deviation angle, wing rotation angle, and pitch torques, respectively. All data is color-coded with the pitch torque-producing wing-beat kinematics (Pitch Torque Modes, see legend in (C)). The doted horizontal lines in (A) and (D) show the wingbeat-average values. **(E)** Wingbeat-average pitch torque versus stroke dihedral angle for the various pitch torque-producing wing-beat kinematics (color-code, see legend in (C)). **(F)** Definitions of the coordinate system, including the body rotations, wingbeat kinematics angles, and sideways velocity vector *V* used in our CFD simulations. **(G)** CFD simulation visualization of the airflow around a fruit fly flying with the sideways velocity *V* = 1 m/s and a stroke dihedral angle Γ = 7.3° (Pitch Torque Mode = -2). Stroke dihedral is defined as the wingbeat-average stroke angle, as shown in (A). **(H)** The parametric space of our CFD analysis, consisting of sideways speeds, stroke dihedral angles, and corresponding Pitch Torque Modes (color-code, see legend in (C)). Here, each dot shows the values for a single CFD simulation. The red box highlights the simulation shown in panel (G), and the gray box highlights the simulations with a hovering fruit fly (*V* = 0 m/s) and of which the data is shown in **(D–E)**. Here, pitch torques are normalized with the order-of-magnitude of the weight and size of an average fruit fly.

To fly, insects beat their wings back and forth using complex wingbeat patterns (Figure 1), whereby they rotate the wing along a wing stroke angle, wing deviation angle, and wing rotation angle. To adjust the aerodynamic forces and torques for flight control, insects primarily alter these wingbeat kinematics by changing the wingbeat frequency, stroke amplitude, stroke dihedral, and the wing angle of attack (Blondeau, 1981; Lehmann and Dickinson, 1998; Taylor, 2001; Ristroph et al., 2013; Muijres et al., 2014; Dickinson and Muijres, 2016; Phan and Park, 2019). Here, we define stroke amplitude as the difference between maximum and minimum stroke angle in a wingbeat, and stroke dihedral as the average stroke angle during the wingbeat (Figures 1A,G).

Moreover, changes in the flight trajectory can be attained by the displacement of an insect’s center of mass (CoM) relative to the center of lift provided by the wings. Such shifts in the animal’s center of mass caused by abdominal motions were observed in the hawkmoths (Sun, 2014). In response to headwind gusts, fruit flies decrease the magnitude of stroke positional angle at ventral stroke reversal, delay wing rotation and make the deviation angles in upstroke and downstroke closer (Gu et al., 2020). These control actions cause rapid gust responses of fruit flies against headwind perturbations. Chirarattananon et al. (2017) performed a set of experiments with a flapping wing robotic insect encountering frontal and lateral drag. The research concludes that the modulation of the wing kinematics in the presence of a disturbance may indirectly alleviate the effect of a wind gust. Interestingly, in birds, wings can act as a suspension system, reducing the effects of vertical gusts by elevating rapidly about the shoulder (Cheney et al., 2020). In lateral gusts, lovebirds actively twist their neck not only to stabilize their visual and inertial head orientation but also to compensate gusts by inferring the local wind direction, while aerodynamic torques rotate their body into the gust (Quinn et al., 2019).

Studying insect flight can lead to the development of more efficient control and design of flapping-wing micro aerial vehicles (MAVs). Furthermore, implementing insect strategies on robots can bring new insights into the flight control of animals that could not be evaluated with animal flight experiments alone. For instance, (Karásek et al., 2018), takes under the scope rapid bank turns observed in escaping fruit flies with the use of a robotic flapper, the DelFly Nimble. A detailed analysis of evasive maneuvers revealed coupling between roll and pitch torques giving an origin to passively induced yaw moments.

In this paper, we combine CFD analysis of sideways flying fruit flies with experiments on freely-flying robots in a side-wind to study the coupling effects between wind-gust-induced aerodynamic forces and the dihedral asymmetry in the wingbeat kinematics. We hypothesize that the coupling between the wingbeat dihedral angle and lateral motion of the flyer results in yaw and roll rotations that passively steer a flapping flyer into the direction of an incoming air gust. Most recent investigation of free-flying lovebirds maneuvering through lateral gusts and experiments on suspended mechanical flapping bird have shown i. a. a similar effect of a side-wind on yaw dynamics. In contrast, our study includes CFD simulations of fruit flies and verifies its findings with free flight experiments of a fully autonomous flapping wing micro air vehicle. In both the CFD simulations and robot experiments, we systematically varied the wingbeat dihedral angle and lateral winds to test this hypothesis and developed simplified models for the observed passively coupled yaw and roll dynamics.

Passively steering a flying device into the direction of a gust can be an effective, effortless, and fast strategy to stabilize a flapping flyer in a windy environment. Many natural flyers flap their wings with a systematic backward-oriented dihedral angle, and this study might help explain why they do so. Furthermore, a similar approach could be used to make flapping robots more gust-resistant. This might be particularly relevant for robots that need to operate in unsteady wind conditions, such as when searching for hazardous gas leaks (Duisterhof et al., 2021). Gas seeking could be complemented not only by sensors installed on a drone but also by using a mechanism such as that presented in this article. During an exploration, in case of an event of gas leakage, the drone would turn into the direction of a source of a gas flow by means of a passive yawing. Detection of the draft followed by measurements from onboard sensors would allow confirming the type of emission, sending further information to the control station and alarm operators in the area, and as a result, preventing a dangerous situation from occurring.

## 2 Fruit fly Flight Kinematics and Control

During hovering flight, fruit flies move their wings back and forth in a horizontal stroke plane. During the wing-stroke, the wing operates at an almost constant angle of attack, approximately 45° (Dickinson and Muijres, 2016). At the end of each half stroke, the wings deflect upward. At the downstroke, the wingtip path is higher than an upstroke’s creating a slight loop ventrally (Ellington, 1999). It is important that during steady hovering flight, the wing is located more behind the body than in front of it. As a result, hovering fruit flies have a positive stroke dihedral angle of approximately 3°. Here, we define the dihedral angle as the wingbeat-average stroke angle. This positive stroke dihedral angle might help to stabilize flight in sideways flight (Karásek et al., 2018).

Fruit flies regulate throttle by adjusting the amplitude and frequency of the stroke. They generate roll moment by enlarging the stroke amplitude of one wing relative to the other, advancing the rotation of that wing, and raising its path. The other wing performs opposite alterations of the wingbeat kinematics. Similar to roll torque, the yaw torque is produced *via* asymmetrical adjustments in the stroke kinematics between the two wings. The main contribution to yaw torque generation has a difference in the wingbeat-average rotation angle of both wings. The angle of attack of one wing is increased, and it is decreased on the contralateral wing. Pitch control is mainly associated with changes in the stroke angle (Figure 1). Wings either flap more forward or backward, resulting in a change in the stroke-dihedral angle and thereby relocating the stroke-averaged center of pressure relative to the fly center of mass (Dickinson and Muijres, 2016).

The adjustments in stroke-dihedral angle might strongly affect the flight stability in sideways wind gusts. Therefore, we will here use the pitch control dynamics to investigate how fruit flies maneuvering in various sideways wind conditions might be stabilized during flight.

CFD simulations solving the Navier–Stokes equations and quasi-steady models that approximate aerodynamic forces on the flapping wings have shown that an insect body pitch is subject to divergent oscillations (Sun and Xiong, 2005; Sun et al., 2007; Faruque and Humbert, 2010; Cheng and Deng, 2011; Ristroph et al., 2013). During hovering flight, the average lift points upwards and counters the gravity force. The horizontal drag for each half-stroke points in the opposite direction. Thus, for a full stroke, we observe the cancellation of the drag forces. Assuming a situation when the insect is pitched forward, the tilted lift force would cause a forward motion of the insect. This creates a net drag acting on the wings and initiates a nose pitch torque, rotating the insect backward. The amplitude of this repetitive movement increases with time. Consequently, this instability may drive the animal to lose control (Ristroph et al., 2013).

Ristroph et al. (2013) presents a theory that describes two strategies to balance the fruit flies in body pitch in flight: active control with sufficiently rapid reactions and passive stabilization with high body drag. Bergou et al. (2010) compares fruit flies wing hinge to a torsional spring that passively resists the wing’s tendency to flip. The spring rest angles can be asymmetrically altered using only a slight active actuation. The created asymmetric rowing motions of the wings induce in-flight sharp turns. Changes in the heading are mostly executed through bank turns (Van Breugel and Dickinson, 2012; Muijres et al., 2014, 2015), thus including not only yaw but also roll or pitch maneuvers. Even though, fruit flies are fully capable of turning by only adapting yaw torque [(Fry et al., 2003; Bergou et al., 2010)].

Presence of a lateral air gust will displace a flyer and will cause it to sideslip. A flying animal can reduce this sideslip by performing a yaw rotation and minimizing or canceling the displacement by rolling into the wind. This can be achieved actively (by wing kinematics adjustments) or passively as a result of passive stability mechanisms of the flapping flyer. Here, we studied how fruit flies flying sideways produce yaw and roll torques to stabilize or destabilize their flight. We did so by performing CFD analyses on sideways-flying in-silico flies (Figure 1). We tested how sideways speed and stroke dihedral angle affect these yaw and roll torques by systematically varying these and running CFD simulations at each combination of stroke dihedral and sideways speed. The dihedral angle was varied by adjusting the wingbeat kinematics of the flies as how they naturally do when adjusting their pitch torque production (Muijres et al., 2014). Finally, based on the results of these simulations, we developed a mechanistic model of the yaw, roll, and pitch torques resulting from the coupling between sideways speed and stroke dihedral angle.

## 3 Materials and Methods

### 3.1 DelFly Nimble

Although natural flapping fliers can vary orders of magnitude in size and their wingbeat kinematics and morphology can differ strongly, the underlying flight dynamics of these flappers can be surprisingly similar (Hedrick et al., 2009; Karásek et al., 2018). Consequently, it is possible to mimic maneuvers of fruit flies with the flapping wing MAV and based on that draw a comparison with the fruit fly data (Karásek et al., 2018). Here, we combined CFD analyses of in-silico fruit flies with experiments on the DelFly Nimble to study the flight dynamics of both natural and artificial flapping flyers in windy conditions, and thereby characterize their generalized flight stabilization mechanisms.

**FIGURE 2:**
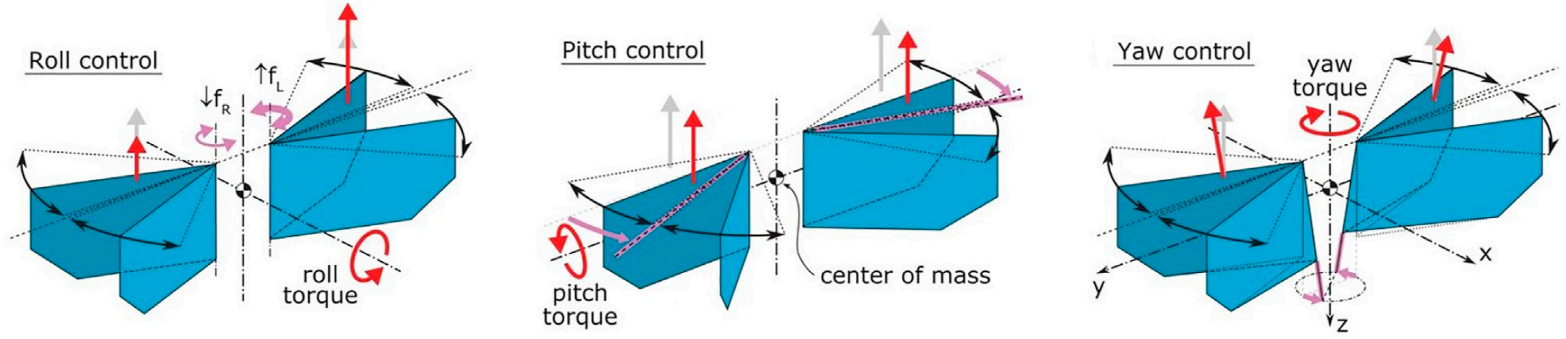
Wing actuation and aerodynamic forces and torques during the roll, pitch, and yaw control. Magenta arrows show actuation action, gray arrows show the nominal wingbeat-average aerodynamic thrust vectors, and red arrows show wingbeat-average thrust and torques after control actuation (adapted from Karásek et al. (2018)).

The DelFly Nimble is an insect-inspired tailless flapping-wing MAV (Karásek et al., 2018). It can hover and fly in any direction (up, down, forward, backward, and sideways). The robot can fly forward with a maximum speed of 7 m/s and sideways with a maximum speed of 4 m/s. The robot is controlled through insect-inspired adjustments of motion of its two pairs of flapping wings (Figure 2). The thrust magnitude is regulated by symmetric modulation of flapping frequencies of the two wings. The pitch torque is produced through adjustment of the dihedral angle, which shifts the wing thrust vector relative to the center of mass. Roll torque is generated by deferentially regulating the stroke frequency of the left and right wing pair. Finally, the yaw torque is produced by changing the wing root angles of the left and right wing pair, such that the wingbeat-average thrust vectors of the wings are tilted in opposite directions with respect to each other. The resulting exceptional agility allows it to perform roll and pitch flips and mimic various insect-inspired maneuvers (Karásek et al., 2018).

### 3.2 Yaw Dynamics Model

For our study, we developed a simplified yaw dynamics model of the Nimble DelFly in sideways winds. We derive it from the model presented in (Karásek et al., 2018), where it was shown that such model gives good predictions of yaw torques during highly dynamic robotic maneuvers, even though it is based on elementary (quasi)steady aerodynamics formulas. Let’s consider a 2D case, employing the quasi-steady assumption and assuming only the translational force component. The flap averaged wing drag force becomes

$$\bar{D}=\frac{1}{T}\int_{0}^{T}\frac{1}{2}\rho C_{D}\left(\alpha \left(t\right)\right)SU{\left(t\right)}^{2}\;dt,$$ (1)

where *T* = 1/*f* is the wingbeat period, *ρ* the air density, *C*_*D*_ the drag coefficient at the angle of attack *α*, *S* the wing area, *U* the wing airspeed, and *t* time.

The model assumes the robot moving with a constant body speed *U*_b_, using a constant flapping speed *U*_f_ = 2Φ*Rf* and constant angle of attack throughout each downstroke and upstroke. Here Φ is the flapping amplitude, *R* is the distance from the flapping hinge point to the wing’s center of pressure, and *f* is the flapping frequency.

**FIGURE 3:**
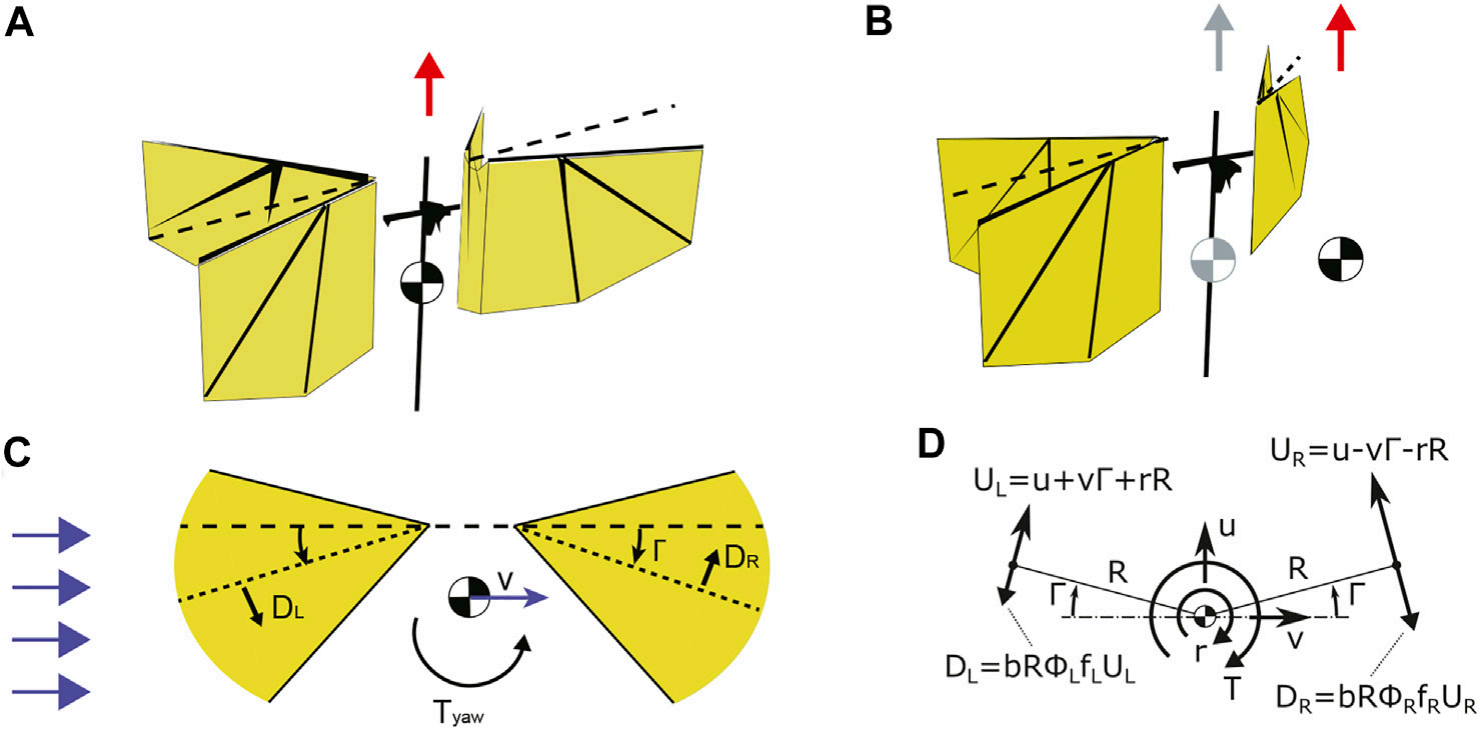
**(A–C)** A drag-based model explaining yaw torque generation due to coupling of sideways velocity with pitch torque generation mechanism *via* dihedral (i.e. mean stroke) angle. **(A)** The red arrow show the wingbeat-average aerodynamic thrust vector of the symmetric configuration of the flyer with center of mass (CoM) positioned at the fuselage. **(B)** The displacement of CoM causes shift in the wingbeat-average thrust (the red arrow) in the direction of the displacement. Shaded color indicates the nominal position of CoM and nominal thrust vector. **(C)** We hypothesize that by introducing a non-zero sideways wind speed (blue horizontal arrows), the body velocity *v* in the lateral body direction will generate yaw torque *T*_*yaw*_ that scales linearly with both sideways speed and dihedral angle Γ. The resulting yaw torque will steer the flapper into the direction of incoming airflow, making the flapping flyer passively stable in the presence of sideways wind. **(D)** Top view diagram showing the effect of the damping forces due to body motion in the horizontal plane on the yaw dynamics. From Karásek et al. (2018).

Satisfying condition *U*_f_ > *U*_b_ (see Figure 3C) and considering the above assumptions we can rewrite Eq. 1 as

$$\bar{D}=\frac{1}{2}{\bar{D}}_{\text{down}}+\frac{1}{2}{\bar{D}}_{\text{up}},$$

$$=\frac{1}{4}\rho C_{D}\left(\alpha \right)S{\left(U_{\text{f}}+U_{\text{b}}\right)}^{2}-\frac{1}{4}\rho C_{D}\left(\alpha \right)S{\left(U_{\text{f}}-U_{\text{b}}\right)}^{2},$$

$$=\rho C_{D}\left(\alpha \right)SU_{\text{f}}U_{\text{b}}=2\rho C_{D}\left(\alpha \right)S\Phi RfU_{\text{b}}=b\Phi fU_{\text{b}}.$$ (2)

The derived drag can be interpreted as a linear damping force, proportional to the body speed *U*_b_, the flapping amplitude Φ and flapping frequency *f*, where the parameter *b* depends on the wing geometry and kinematics. The normal component of the speed due to body motion can be identified for the left and right wing pair as

$$U_{\text{L}}=v\Gamma +rR,$$

$$U_{\text{R}}=-v\Gamma +-rR,$$ (3)

where Γ is the dihedral angle (since Γ < 25° we use the approximation sin Γ ≈ Γ), *u* and *v* the body velocity in the longitudinal and lateral body direction and *r* is the angular rate around the yaw axis of the body.

The yaw torque acting on the body can be expressed as

$$T=R\left(D_{\text{R}}-D_{\text{L}}\right).$$ (4)

The drag forces of the individual wing pairs can be obtained by substituting the speed in Eq. 3 for *U*_b_ in Eq. 2, giving the solution for the yaw torque as

$$T=-bR\left[\left(f_{\text{R}}\Phi_{\text{R}}+f_{\text{L}}\Phi_{\text{L}}\right)\left(Rr+\Gamma v\right)\right],$$ (5)

which can be easily broken down into two components

$$T=T_{\text{FCT}}+T_{\text{yaw}}$$ (6)

where *T*_*FCT*_ is the flapping-counter-torque

$$T_{\text{FCT}}=-bR^{2}\left(f_{\text{R}}\Phi_{\text{R}}+f_{\text{L}}\Phi_{\text{L}}\right)r,$$ (7)

*T*_yaw_ is a yaw torque that scales linearly with both sideways speed and dihedral angle.

$$T_{\text{yaw}}=-bR\left(f_{\text{R}}\Phi_{\text{R}}+f_{\text{L}}\Phi_{\text{L}}\right)\Gamma v.$$ (8)

### 3.3 Studying the Flight Dynamics of a Freely-Flying MAV in Sideways Winds

Here, we studied how a freely-flying flapping MAV behaves hovering in sideways winds, and how a wing stroke dihedral affects this. Inspired by the clap-and-fling mechanism observed in nature, two wings on each side of the robot flap in counter-phase and clap and peel with each other to enhance the produced thrust. The thrust magnitude is regulated by symmetric modulation of flapping frequencies of both wings. By displacing the center of mass as shown in Figure 3B we can shift the thrust vector in the direction of the displacement, hence causing a change in the dihedral angle Γ. We hypothesize that by introducing a non-zero sideways wind speed, the body velocity in the lateral body direction will generate *T*_yaw_ that scales linearly with both sideways speed and dihedral angle (Figure 3C). The resulting yaw torque will steer the flapper into the direction of incoming airflow, making the flapping flyer passively stable in the presence of sideways wind by reducing a sideslip. The induced yaw rotation and thus heading change take place without substantial course change.

### 3.4 CFD Analysis of Fruit Flies Flying in a Side-Wind

We performed a systematic CFD study by performing CFD simulations of fruit flies flying at various sideways flight speeds and with a range of wing-stroke dihedral angles (Figure 1H). The sideways speeds ranged from 0 to 1 m/s in steps of 0.1 m/s. We tested a range of five wing-beat kinematics with wing-stroke dihedral angles of 0.8, 2.4, 4.0, 5.7, and 7.3° (Figures 1A–D). These wing-beat kinematics are those used by fruit flies to produce various pitch torques (Muijres et al., 2014), ranging from high pitch-down torques to high pitch-up torques (Figure 1E). Each kinematics case is defined by both the Pitch Torque Mode number (-2, -1, 0, 1, and 2) and the corresponding stroke dihedral angle (Figure 1E), and is reconstructed from the wing-beat kinematics modulations in Muijres et al. (2014).

We performed a CFD simulation for each combination of sideways speed and stroke-dihedral angle (Figure 1H), resulting in 55 simulations in total. For each simulation, we simultaneously accelerated the body sideways until it reached the desired sideways speed and accelerated both the wings from standstill to their cases-specific wing-beat kinematics (Supplementary Movies S1–S6). We then simulated four wingbeats at that constant sideways speed; we used the final one of these wingbeats for our analysis. The flight kinematics is defined using an aerospace coordinate system in the body reference frame (Figure 1F). Note that we enforced the body and wing kinematics, and thereby ignore any damping effect of the flexible free-flying animal.

#### 3.4.1 CFD Simulation Setup

All CFD simulations were performed using the software IBAMR (Bhalla et al., 2013). It is an immersed-boundary adaptive mesh refinement code that lends itself well to studying flapping flight. The solver has been tested and was optimized for studying fruit fly flight in a previous study, by comparing CFD simulation results with those from a dynamically-scaled robotic flapper experiment (van Veen et al., 2019). The optimization process consisted of a grid refinement study and a time-step study. Based on this, we performed all our experiments at a temporal resolution of Δ*t* = 1 × 10^−7^s and a baseline spatial resolutions of Δ*x* = 0.01 mm. We used adaptive mesh refinement based on the vorticity in the flow field with three mesh refinement levels. The corresponding vorticity thresholds of 50  s^−1^, 500  s^−1^, 5,000  s^−1^ from coarsest to finest, respectively. The simulations were run with a maximum Courant–Friedrichs–Lewy (CFL) value of 0.5, though this value was never reached; the mean CFL of all simulations was around 0.01.

#### 3.4.2 Analyzing the CFD Data

For each simulation, we analyzed the aerodynamics of the last simulated wing-beat. We determined the temporal dynamics of the aerodynamic forces and torques produced by the fruit fly during that wing-beat, directly from the constraint force term of the no-slip boundary condition at the wing and body surfaces (Bhalla et al., 2013). In addition, we visualized the corresponding airflow dynamics using vorticity iso-surfaces, color-coded with the air-pressure coefficient (Figure 1G). From the temporal dynamics of the aerodynamic torques, we determined the wing-beat-average forces and torques. We used the resulting wing-beat-average roll, pitch, and yaw torques to test how these varied with sideways speed and wing stroke dihedral angle. We normalized all forces with the approximate average weight of a fruit fly (*W* = 1 mg), and all torques with the product of this weight and the size order of a fruit fly (1 mm).

#### 3.4.3 Modeling Torque Coupling in Sideways Flying Fruit Flies

**FIGURE 4:**
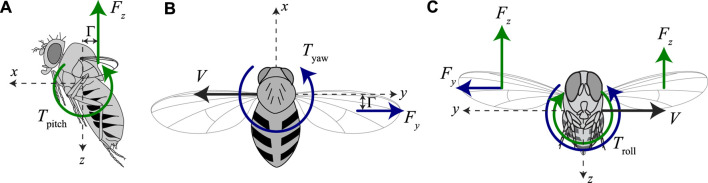
Our proposed body torque model for fruit flies flying sideways at variable speeds and with variable pitch torque-producing wing-beat kinematics. **(A–C)** Free body diagrams of fruit flies flying sideways, including the pitch, yaw, and roll torque model, respectively. **(A)** Pitch torque equals the product of the vertical aerodynamic force (|*F*_*z*_|) and the stroke dihedral angle, as proxy for the pitch torque moment arm. **(B)** Yaw torque equals the product of the sideways aerodynamic drag force (*F*_*y*_) and the stroke dihedral angle, as proxy for the yaw torque moment arm. **(C)** Roll torque depends on both the sideways aerodynamic drag force (*F*_*y*_) and the asymmetry in vertical aerodynamic force of the left and right wing (Δ*F*_*z*_). These are assumed to scale linear and quadratic with sideways flight speed, respectively.

Based on the torque model developed by Karásek et al. (2018) and extended in Section 3.2, we propose a simplified functional model of roll, pitch, and yaw torque production by sideways flying fruit flies, with variable wing stroke dihedral angles, and variable aerodynamic force production (Figure 4).

Our model assumes that pitch torques scale with the product of the vertical aerodynamic force magnitude and the stroke dihedral angle, as a proxy for the pitch torque moment arm (Figure 4A). With this linear model, we thus assume that pitch torque is independent of sideways speed. To remove the effect of aerodynamic force magnitude on pitch torque, we here define our normalized pitch torque as

$$T_{\text{pitch}}^{*}=T_{\text{pitch}}/|{F_{z}}_{\text{pitch}}|l$$ (9)

where *T*_pitch_, $\left|{F_{z}}_{\text{pitch}}\right|$ and *l* = 1 mm are pitch torque, the absolute vertical force and the average length scale of a fruit fly, respectively. According to our model, this normalized pitch torque should scale with stroke dihedral as

$$T_{\text{pitch}}^{*}=K_{\text{pitch},\Gamma }\Gamma +K_{\text{pitch}}$$ (10)

where *K*_pitch,Γ_ and *K*_pitch_ are the pitch torque coefficients, and Γ is the wing stroke dihedral angle. Here, *K*_pitch_ equals the normalized pitch torque at zero stroke dihedral.

Secondly, we propose that yaw torques scale linearly with the product of the sideways drag force and the stroke dihedral angle, as a proxy for the yaw torque moment arm (Figure 4B). The yaw dynamics model in Section 3.2 shows that the sideways drag force scales linearly with the sideways speed, and thus our model becomes

$$T_{\text{yaw}}=K_{\text{yaw}}\Gamma V_{y}$$ (11)

where *V*_*y*_ is the sideways speed of the fly, and *T*_yaw_ and *K*_yaw_ are the yaw torque and corresponding coefficient, respectively.

Finally, roll torques depend on both the sideways aerodynamic forces and the difference in upward forces produced by both wings (Muijres et al., 2014) (Figure 4C). Our model predicts that the sideways forces produced by the beating wings scale linearly with the sideways speed, whereas the upward forces scale with airspeed squared. Therefore, we propose here a quadratic torque model as

$$T_{\text{roll}}=K_{\text{roll},Fy}V_{y}+K_{\text{roll},Fz}V_{y}^{2}/F_{z}$$ (12)

where *T*_roll_ is roll torque, *K*_roll,*Fy*_ is the coefficient for roll torques resulting from the sideways forces, *K*_roll,*Fz*_ is the coefficient for roll torques resulting from the upward aerodynamic forces. The normalization with the vertical aerodynamic force is done to control for variations in upward forces, independent of the effect of sideways speed.

We tested these models by fitting the simulation results to each model using non-linear least squares curve fitting in Matlab (Mathworks Inc.). From this fitting, we have determined the goodness of fit and torque coefficients.

### 3.5 Flapping Wing MAV Experiments

#### 3.5.1 MAV Experimental Setup

**FIGURE 5:**
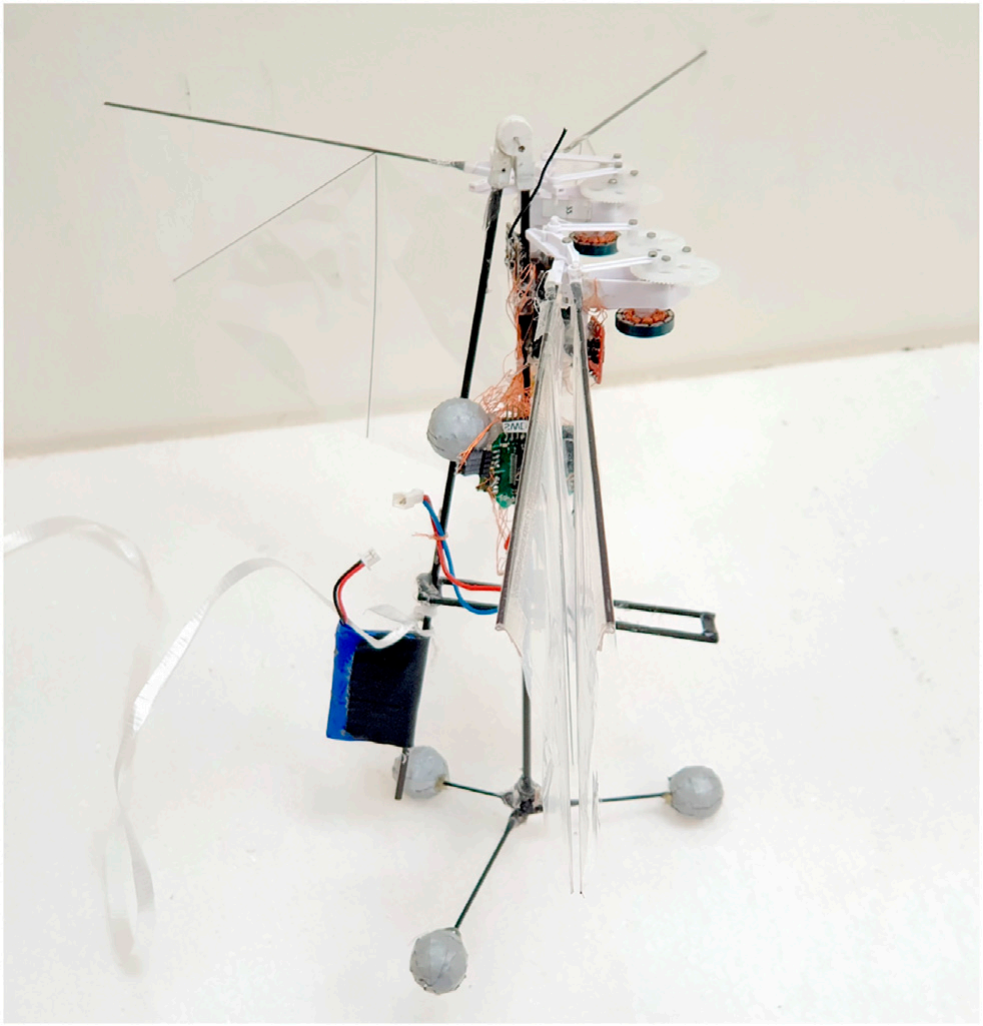
The asymmetric flapping wing MAV used in the experiments, equipped with reflective markers of the motion tracking system and a new divider-like mass displacement mechanism.

The flapping wing MAV used in the experiments is an altered version of the DelFly Nimble. The robot design is expanded by a new divider-like mass displacement mechanism, further explained in the next subsection. The flapper is equipped with an open-source STM32F4-based Lisa/MXS autopilot for active attitude stabilization. The board is running an open-source Paparazzi UAV autopilot system. The board features a 72 MHz ARM Cortex-M3 microcomputer and an MPU6000 6-axis MEMS IMU consisting of a 3 axis gyroscope and accelerometer among other sensors. The attitude is stabilized by a standard PD controller with additional low-pass filtering. The data link between the autopilot and the ground station is obtained *via* the ESP8266 ESP09 WiFi module. This was invaluable during the testing, as it provided live telemetry and allowed online tuning of the various control parameters. To sustain a 4–5 min long flight, depending on the complexity of maneuvers, the robot requires a 180 mAh, 3.7V LiPo battery. To perform an analysis of the onboard power consumption, the ASC711 Hall effect-based linear current sensor was added to the autopilot board. The final vehicle configuration, with a total mass of 34.5 g is shown in Figure 5.

#### 3.5.2 A Divider-Like Mass Displacement Mechanism

**FIGURE 6:**
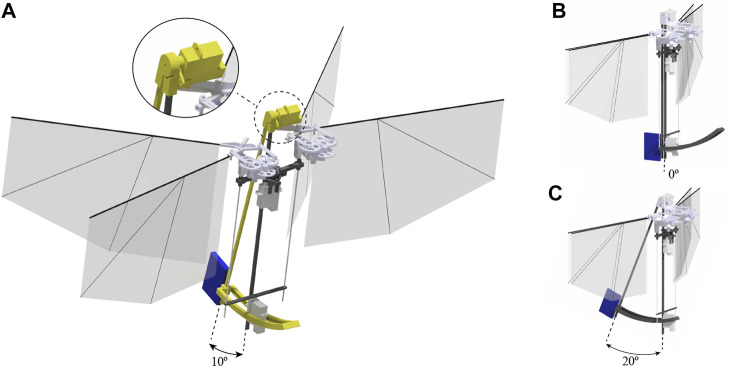
Mass displacement mechanism (colored in yellow) of the asymmetric flapping wing MAV allows setting the desired displacement angle from 0 to 20°. The mass displacement of 0 cm, 2 cm, and 4 cm is corresponding to displacement angle of the mechanism of **(B)** 0° **(A)** 10° **(C)** 20° accordingly. The battery is accounting for 17% of the mass and is marked in blue.

To study our hypothesis on the robot, we designed a new divider-like mass displacement mechanism, shown in Figure 6. The battery of the robot is accounting for 17% of its total mass, being the heaviest part of the flapper. Thus, to adjust the center of mass (CoM), we adjust the position of the battery itself. The developed mechanism consists of a carbon rod with the battery attached to its bottom and a 3 days printed hinge connecting the mechanism with the fuselage. A servo motor is controlling the rotation of the hinge and through that the displacement angle of the system. Carbon fiber rails at the bottom prevent the sideways motion of the designed mechanism with respect to the fuselage. The setup can be controlled actively by adjusting the displacement angle *via* a command sent from the transmitter. In the interest of having s stable displacement angle without additional vibrations caused by the servo actuator, during the experiments we are not using this functionality. Instead, fixing it to the desired angle mechanically.

#### 3.5.3 Test Setup

**FIGURE 7:**
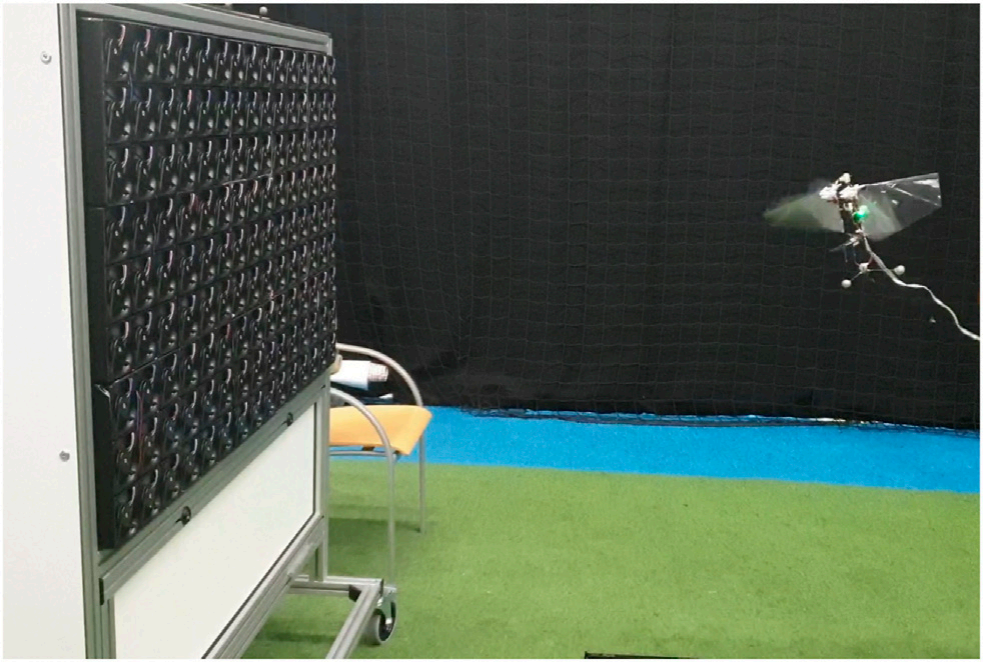
The test setup of free flight experiments of the asymmetric flapping-wing MAV with the low-speed multi-fan wind system.

To test our new design we performed free-flight tests in the Cyberzoo, a flight arena (10 m × 10 m × 7 m) of TU Delft equipped with a motion tracking system consisting of 12 OptiTrack Prime 17 W cameras. A low-speed multi-fan wind system (Olejnik et al., 2022) was placed in the arena, which allowed to test the MAVs responses to wind gusts (Figure 7). The wind system consists of an array of 135 axial fans (8412N/2GHP ebm-papst) that occupy a space of 1.2 × 0.75 m, for a total wind surface of 0.9 m^2^. All the processing of the robotic experiments was carried out using MATLAB 2020b software (MathWorks, Inc.). The motion tracking and the onboard logged data were synchronized and the analysis of the gathered data is presented in results section.

During the experiments, the robot is flying into the waypoint located in front of the wind system at 1 m distance and 1 m height, gathering data about states, battery voltage, and current. The yaw control of the MAV is turned off except in the baseline case to which we compare experimental data. Because the yaw actuator is never trimmed perfectly, without feedback control a small amount of yaw torque is generated. (Thanks to the Flapping Counter Torque), this results in a slow, steady yaw rotation. The operator of the wind system waits till the orientation of the robot is perpendicular with the respect to the front of the wind system corresponding to 0° yaw angle. Next, the wind speed is set to the desired value ranging from 0 to 1.5 m/s, and we can observe an immediate response of the MAV, which aligns its heading with the wind direction. We repeat the experiment for three different configurations of the robotic flyer; with the mass displacement of 0 cm, 2 cm, and 4 cm corresponding to the divider-like mechanism’s displacement angle of 0°, 10°, 20° accordingly.

## 4 Results and Discussion

Our study consisted of a set of CFD simulations on in-silico flying fruit flies (Supplementary Material S1), and a set of experiments with a bio-inspired robotic flyer. Here, we discuss first the results of these two topics separately, followed by the integrated result of the robot and CFD combined.

### 4.1 Sideways Flying Fruit Flies

We reconstructed the wingbeat kinematics exhibited by fruit flies to produce various amounts of body pitch torques (Figures 1A–C) (Muijres et al., 2014). The stroke dihedral angle of these wingbeats, defined as the wingbeat-average stroke angle, varies from close to zero degrees to almost 8° between the five pitch-torque modes. The CFD simulations with hovering fruit flies exhibiting these five pitch-torque modes (Supplementary Movies S1–S3) show that body pitch torques scale close to linearly with stroke dihedral (Figure 1E). During hovering flight, the corresponding roll and yaw torques are per definition zero for all tested kinematics patterns, because these wingbeat kinematics are symmetrical between the left and right wing.

**FIGURE 8:**
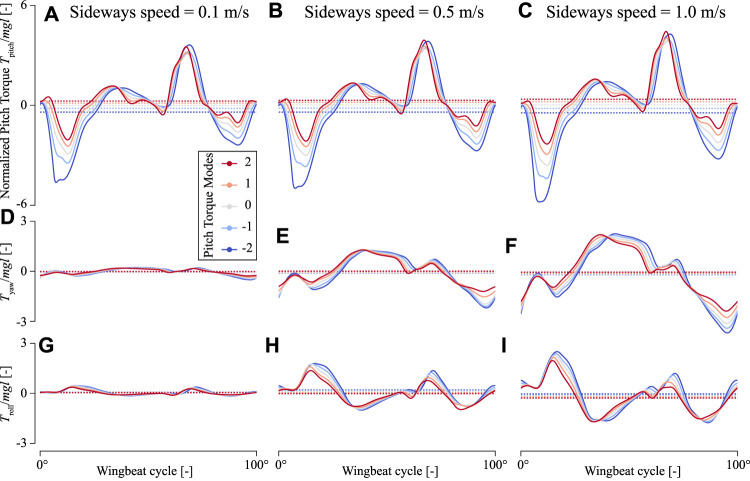
Temporal dynamics of the torques produced by an in-silica fruit fly flying sideways with variable pitch torque-producing wing-beat kinematics. **(A–C)** Pitch torques **(D–F)** yaw torques, and **(G–I)** roll torques, respectively. The first to last columns show torques produced at a sideways flight speed of 0.1, 0.5, and 1.0 m/s, respectively. Each panel shows torques determined using CFD (ordinate) throughout a single wingbeat cycle (abscissa) for the various pitch torque-producing wing-beat kinematics (color-code, see legend). The doted horizontal lines show the wingbeat-average torques. All torques are normalized with the order-of-magnitude of the weight and size of an average fruit fly.

**FIGURE 9:**
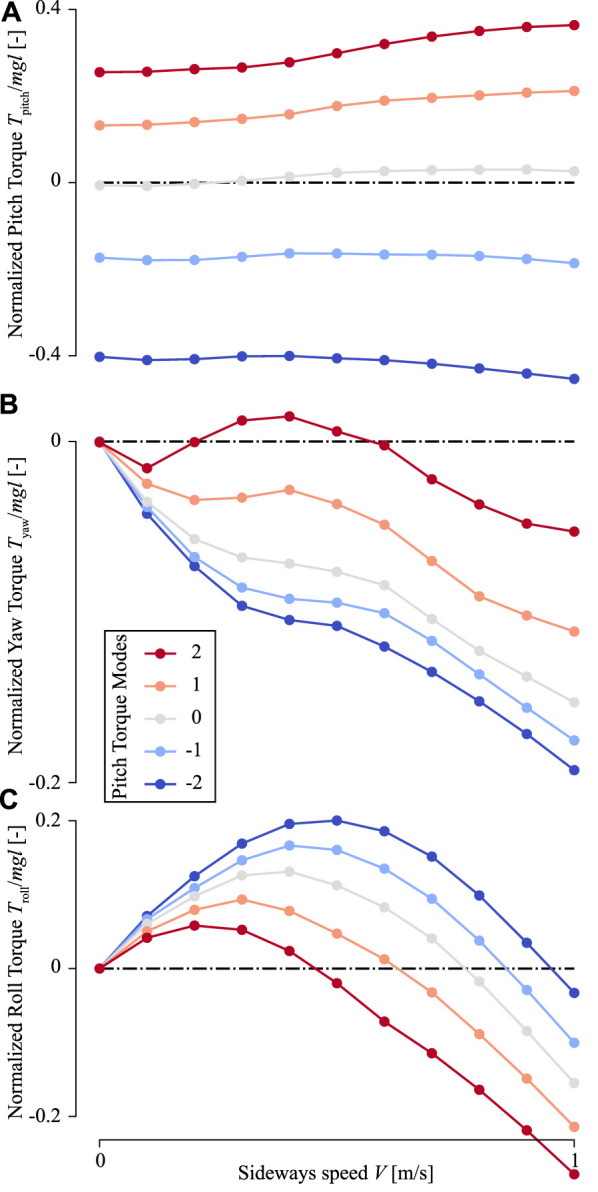
The wing-beat-average torques produced by an in-silica fruit fly flying sideways with variable pitch torque-producing wing-beat kinematics. **(A–C)** Pitch, yaw, and roll torques, respectively. Each panel shows torques determined using CFD (ordinate) as a function of sideways flight speed (abscissa) and wing-beat kinematics (color-code, see legend). Each data point shows the wing-beat-average torque resulting from a single CFD simulation. All torques are normalized with the order-of-magnitude of the weight and size of an average fruit fly.

In sideways flight, the roll and yaw torques produced by the in-silico fly are non-zero, and these torques increase with sideways speed (Figures 8D–I). In contrast, the pitch torques are affected relatively little by sideways speeds (Figures 8A–C). The temporal dynamics of these pitch torques throughout a single wing beat show that peak pitch torques do increase slightly with increasing sideways speeds, but the positive and negative torques do so similarly. As a result, these positive and negative torque peaks mostly cancel each other, resulting in a relatively small effect of sideways speed on wingbeat-average pitch torques (Figure 9A). Therefore, the effect of variations in stroke dihedral angle dominates in the variations in pitch torque dynamics, above those of sideways speeds.

**FIGURE 10:**
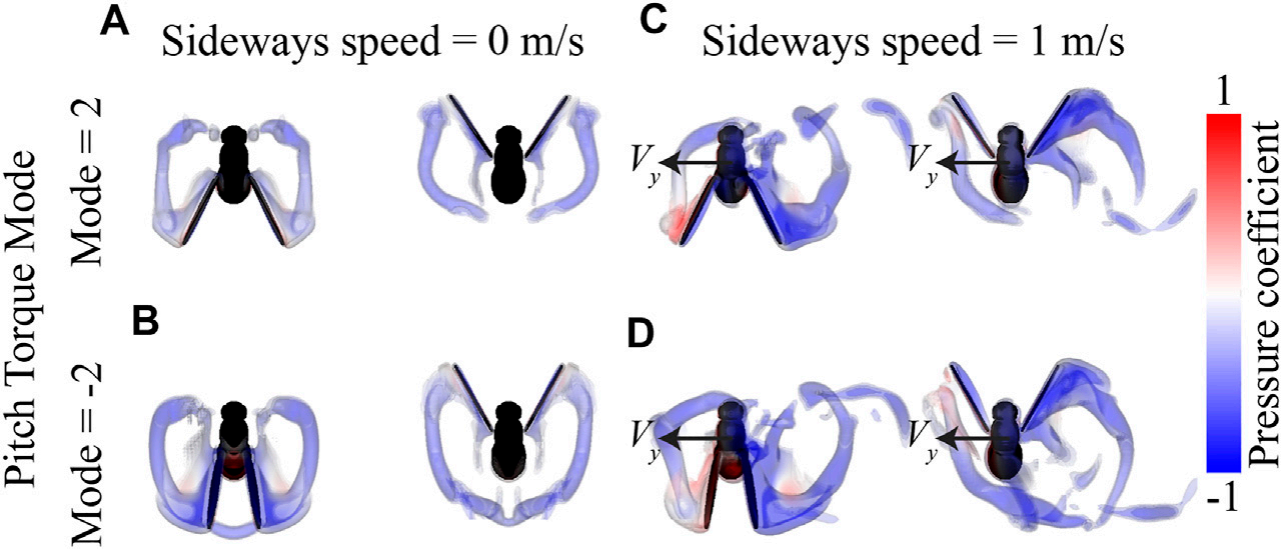
Visualization of the airflow around flying fruit flies determined using CFD simulations. **(A,B)** Airflow visualizations around hovering fruit flies (*V* = 0 m/s), with the wingbeat pattern of Pitch Torque Mode = 2 and -2, respectively. **(C,D)** Airflow visualizations around fruit flies flying with a sideways speed of (*V* = 1 m/s, and Pitch Torque Mode of 2 and -2, respectively. Each panel shows results for the stroke-reversal at dorsal and ventral sides (left and right, respectively). All flies are shown from above, and the airflow is visualized using iso-surfaces of constant vorticity (4,000 and 5,000 s^−1^), color-coded with the air-pressure coefficient at the surfaces (see legend).

In contrast, yaw torque dynamics are affected more by sideways speeds. The temporal dynamics of yaw torques increase strongly with increasing sideways speed, and to a smaller extent with stroke dihedral angle (Figures 8D–F). Here, the sideways speed results in the production of negative yaw torques when the wing is positioned on the dorsal side of the fly, and positive torques when the wing is on the ventral (forward) position. The airflow visualizations around the flying fly highlight the cause of these yaw torque dynamics (Figure 10 and Supplementary Movies S4–S6). During sideways flight, the wing positioned on the leeward side of the fly produces a large low air-pressure region during both ventral and dorsal stroke-reversal. This low air-pressure region results in a wind-induced drag force on that wing, and which has a relatively large yaw torque moment arm. As a result, the fly flying in side-winds generates significant yaw torques, especially during stroke-reversal. These yaw torques are positive and negative during ventral and dorsal stroke-reversal, respectively. Thus, for a close-to-symmetric wingbeat (stroke dihedral close to zero) the positive and negative yaw torques mostly cancel each other (Figure 10C and Supplementary Movie S5), resulting in a negligible wingbeat-average yaw torque in sideways wind. But with increasing stroke dihedral, the dorsal-ventral yaw torque dynamic becomes more asymmetric, causing an increase of negative wingbeat-average yaw torques (Figures 8D–F). As a result, fruit flies flying sideways with hovering or pitch-down wingbeat kinematics produce a small but significant negative wingbeat-average yaw torque (Figures 10C, 9B; Supplementary Movies S4–S6, respectively).

Roll torque dynamics are also affected by sideways speeds (Figures 8G–I). The temporal dynamics of the sideways-speed-induced roll torques have a relatively complex pattern. During the first half of each wing-stroke roll torques are positive, whereas during the second half of the wing-stroke roll torques are negative. This is both the case for the forward wing-stroke and the backward stroke, although torques are higher in the forward wing-stroke.

These dynamics can be explained by the aerodynamic torque model that we developed for fruit flies flying in a side-wind (Eq. 12 and Figure 4). Roll torques produced by a fruit fly flying in a side-wind are caused by both the sideways drag force (*F*_*y*_) and the asymmetry between the upward lift forces produced by the two wings (*F*_*z*_). The sideways drag force is always directed away from the sideways velocity vector *V*_*y*_ and is expected to scale linearly with this speed. The upward-directed force scales quadratically with the airspeed over the wing, and thus also with the sideways speed. But when a wing moves towards the sideways incoming air, the sideways speed increases lift forces on the wing, whereas when the wing moves with the side-wind lift forces on the wing are decreased. As a result, the lift forces on the windward wing are increased during the first half of the wing-stroke (moving into the wind), and lift forces are decreased during the second half of the wing-stroke (windward wing moves with the wind). For the leeward wing, these dynamics are reversed. This asymmetry in lift force production between the windward and leeward wing causes the observed roll torque dynamics (Figures 8G–I and Supplementary Movies S4–S6). As a result of the combined linear and quadratic dependence of roll torque on side-wind speed (Eq. 12), the wingbeat-average roll torque scale also non-linearly with sideways speed (Figure 9C).

**FIGURE 11:**
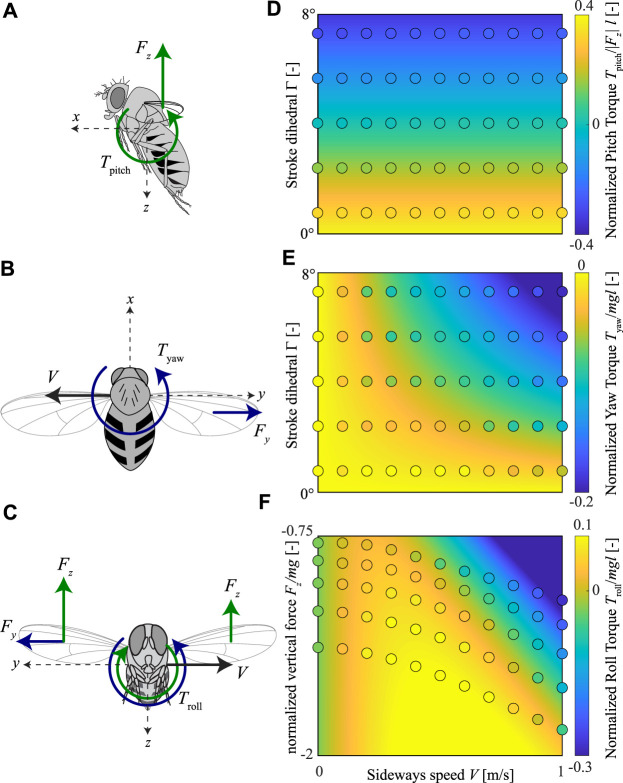
Fit of our proposed body torque model for sideways-flying fruit flies to the CFD data. **(A–C)** Free body diagrams of fruit flies flying sideways, including the pitch, yaw, and roll torques, respectively. **(D–F)** The pitch, yaw, and roll torques, respectively, within the parametric space of all CFD simulations. Each panel shows the normalized torques in colors (see color maps on the right), whereby the CFD simulation results are shown by circles and the model fit is shown as contour plots. The parametric space for pitch and yaw torques consists of sideways flight speed (abscissa) and the stroke dihedral angle (ordinate); for the roll torques, the parametric space consists of sideways flight speed (abscissa) and the vertical aerodynamic force (ordinate). Yaw and roll torques are normalized with the order-of-magnitude of the weight and size of an average fruit fly, whereas pitch torques are normalized by the absolute of the vertical aerodynamic force (|*F*_*z*_|) and the average size of a fruit fly.

We fitted the aerodynamic torque models proposed for fruit flies flying in a side-wind and with non-zero stroke dihedral angle to the wingbeat-average torques determined using our CFD analysis (Eq 10–12; Figures 9, 11A–C, , respectively). The model fits capture the observed wingbeat-average torque dynamics well, as shown by the comparison of the predicted model output with the CFD results (Figures 11D–F). For the pitch torque model, the fitted coefficient values are *K*_pitch,Γ_ = − 0.090 9 ( − 0.092 8, − 0.089 0) and *K*_pitch_ = 0.389 4 (0.380 7, 0.398 2) as mean (confidence interval). The goodness-of-fit parameters are *R* ^2^ = 0.994 and root-mean-square-error rmse = 0.02. For the yaw torque model, the fitted coefficient value is *K*_yaw_ = − 0.031 4 ( − 0.033 4, − 0.029 4), and the goodness-of-fit parameters are *R* ^2^ = 0.865 and rmse = 0.02. Finally, for the roll torque model, the fitted coefficient values are *K*_roll,*Fy*_ = 0.680 2 (0.626 8, 0.733 6) and *K*_roll,*Fz*_ = 1.155 (1.068, 1.241). The corresponding goodness-of-fit parameters are *R* ^2^ = 0.927 and rmse = 0.03. These results combined show that our simplified model captured the pitch, roll and yaw torque dynamics of sideways flying flies well, although some higher-order dynamics are ignored.

Both our CFD data and aerodynamic torque model predict that fruit flies flying sideways should produce stabilizing yaw torques that would turn them into the direction of the wind. Due to the baseline positive stroke dihedral of trimmed hovering fruit flies, these stabilizing yaw torques are also produced when hovering or in slow forward flight. These stabilizing negative yaw torques increase with wing-stroke dihedral angle, and thus flies that produce pitch-down torques simultaneously enhance their yaw-based air gust rejection capabilities. In contrast, when producing rapid pitch-up torques, the sideways-wind-induced yaw-torques are too close to zero.

The roll-torque dynamics are more complex, as roll-torques scale quadratically with sideways wind speed. As a result, at low side winds, roll torques cause the animal to roll away from the incoming side-wind. This causes the animal to accelerate in the same direction as the wind and thereby destabilizing its flight. But due to its non-linear dynamics, the roll torques become negative at high speeds. These negative torques cause the animal to roll into the wind and thus stabilize its roll dynamics (Figures 11C,F). Our model predicts that a fly that produces weight support (*F*_*z*_/*mg* = − 1) produces stabilizing negative roll torques at sideways speeds larger than 0.59 m/s. Thus, flying fruit flies are particularly robust against the most dangerous high-speed wind gusts.

The yaw and roll torque-coupling dynamics suggest that a fly encountering a high-speed sideways wind-gust can respond by only producing a pitch-down torque. When a flying fruit fly pitches down, it redirects the upward-directed aerodynamic force vector forward. This allows the animal to accelerate forward and minimize the gust-induced displacement. Because of the passive yaw torque that reduces sideslip, the fly can perform this pitch-down maneuver in response to both forward and sideways directed wind gusts. At wind gust speeds higher than 0.59 m/s, the roll torques cause the animal to roll into the wind. The yaw torques enhanced by the pitch-down maneuver (increased stroke-dihedral) cause the animal to also rapidly yaw into the wind. The pitch-down maneuver itself causes the animal to further accelerate into the wind, and thereby minimizing drift. We used the experiments with the bio-inspired flapping flying robot to test whether these dynamics also occur in free-flight conditions.

### 4.2 Sideways Flying Robotic Flyers

**FIGURE 12:**
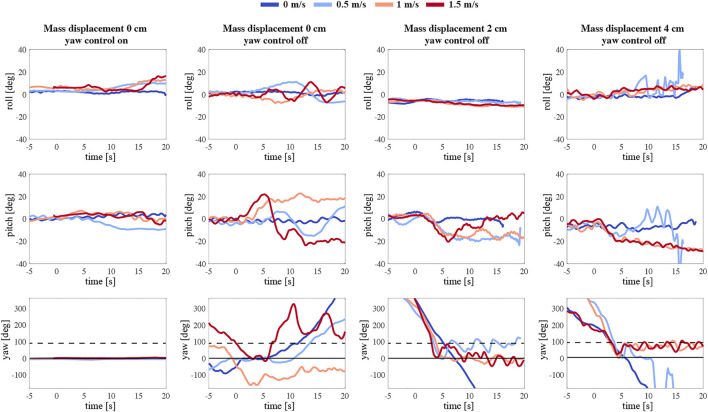
The temporal dynamics of body attitude of the robotic flyer flying at various wind speeds (see legend on top), and with various configurations. The top to bottom rows show body roll, pitch and yaw data, respectively. The first to last column show results for the various configurations, which are a mass displacement of 0 cm and yaw control on and off (first and second row, respectively), and yaw control off with a 2 and 4 cm mass displacement (third and last column, respectively). The solid horizontal black line at 0° yaw angle corresponds to sideways position of the robot with respect to the incoming wind gust, and the black dashed line at 90° yaw angle corresponds to forward flight into the incoming wind gust.

Figure 12 presents the body attitude during free flight tests of the robotic flyer with a mass displacement of 0, 2, ,and 4 cm at various wind speeds. The first column shows the experiments with yaw control enabled, whereas the second through fourth columns show experiments where yaw control was turned off. The timeline corresponds to three phases of the maneuver: First, from −5 to 0 s, the robotic flyer is hovering at the waypoint of 1 m distance from the wind multi-fan system. Once its orientation is perpendicular to the outlet of the wind system corresponding to 0° yaw angle, at around 0 s, the wind speed is set to the desired value ranging from 0 to 1.5 m/s. The response of the flier to the crosswind can be observed further on.

During the first experiment (first, left column in Figure 12), the robot is symmetrical. The motion tracking system assures maintaining the sideways position of the MAV in the wake of the wind system. Its body attitude is controlled in all three axes: roll, pitch, and yaw. The body angles at the beginning of the test are constant and close to 0. While introducing a crosswind the roll angle increases to almost 20° due to the provided extra lift. The wind also affects the pitch axis, causing a deviation in the pitch angle. The flyer sustains a constant 0-degree yaw angle as commanded. Next, we repeat the experiment but with the yaw control turned off (second column). Immediately, we can observe a drift in the robot yaw axis. Once the wind reaches the flapper the instability along all the axes grows. We can see that with higher speeds the MAV turns around and oscillates between its natural equilibrium - facing the wind (Supplementary Movie S7).

Subsequently, we introduced an asymmetry to the MAV by adjusting the mass displacement mechanism, thus shifting the mass below the center of mass backward by 2 cm (third column). At around the fifth second of the free flight, we can observe the influence of the incoming side wind on the robot (Supplementary Movie S8). The flapping flier in this configuration produces a more consequent nose-down pitching moment. Although the robot experiences an immediate yaw torque, the stabilizing effect is rather momentary.

**FIGURE 13:**
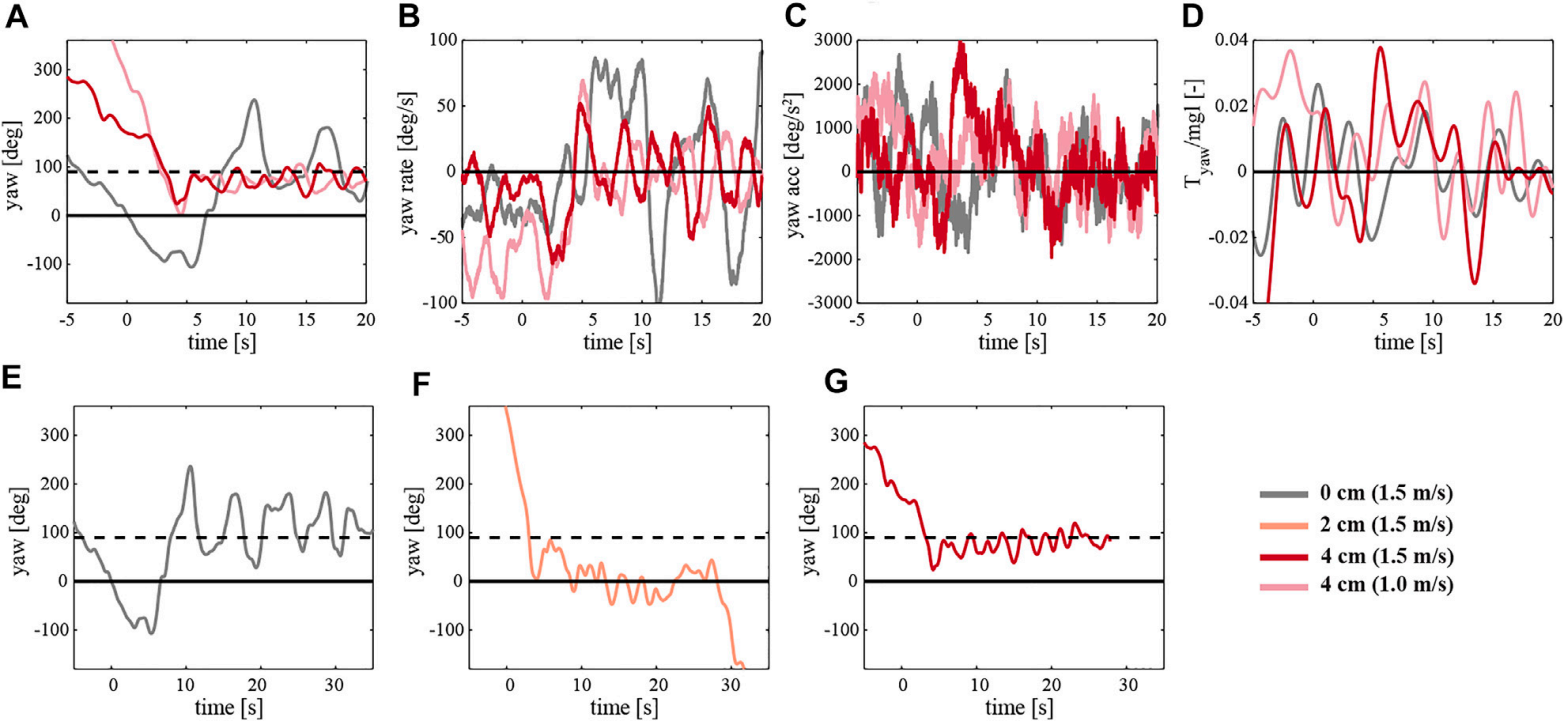
**(A–D)** The temporal yaw dynamics of the robot flying in a side-wind with yaw control turned off, and with various mass displacements. **(A–D)** Yaw dynamics of the robot flying at a 1.5 m/s wind speed and with 0 cm mass displacement (grey), and the robot with a 4 cm mass displacement flying in a 1 m/s and 1.5 m/s wind speed (orange and red, respectively). Yaw dynamics consist of the yaw angle **(A)**, yaw rate **(B)**, yaw acceleration **(C)**, and yaw torque normalized by the weight and size of the robot **(D)**. **(E–G)** The temporal dynamics of the yaw angle of the robot flying without yaw control, at wind speed of 1.5 m/s, and with a mass displacement of 0 cm **(E)**, 2 cm **(F)**, and 4 cm **(G)**. The black line at the 0-degree yaw angle corresponds to the sideways position of the robot and the black dashed line at 90° angle to forward flight in the incoming wind gust.

The final configuration, a 4 cm backward displacement of the mass, best presents the stabilizing effect of the passively induced yaw torque (right column). After a turn of around 90° induced by a side wind of 1 and 1.5 m/s, the robotic flyer continues to keep its body attitude (Supplementary Movie S9). The yaw angle is converging to around 90°, corresponding to forward flight in the incoming wind gust with a substantial pitch angle. Figure 13D shows the development of the yaw torque during the test. A more detailed inspection of the yaw angle convergence time after the turn reveals a clear benefit of the adopted asymmetric design of the robot. Figures 13E–G compares the 4 cm case with the 0 and 2 cm case at the wind speed of 1.5 m/s. The body attitude in the yaw axis needs around 30 s to reach a steady state, whereas the 4 cm asymmetric configuration only needs 2 s; curiously, the intermediate 2 cm asymmetric configuration performed poorest.

**FIGURE 14:**
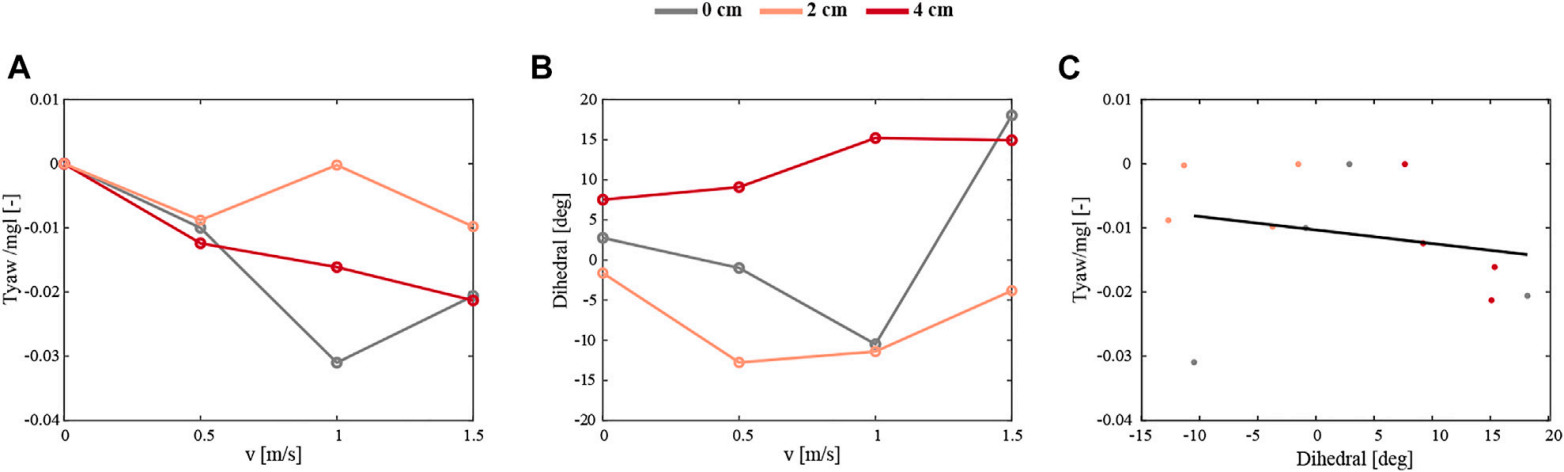
The average yaw torques and dihedral angles gathered during 5 s of flight experiments with the robot flying without yaw control in a side-wind. The 5 s time window correspond to the moment that the robot transitioned from sideways flight to forward flight in the wind gust. **(A,B)** Yaw torques **(A)** and dihedral angles **(B)** as a function of sideways velocity for the robot flying with a mass displacement of 0 cm, 2 and 4 cm (in grey, orange and red, respectively). **(C)** Yaw torques versus dihedral angle for the same cases as shown in **(A,B)**. The solid black line shows a linear fit through all data combined.

To compare the robotic experiments to CFD studies of fruit flies, we look into the relation of the dihedral angle and yaw torque with the sideways velocity (Figure 14). This shows that the sideways flying robot produced stabilizing negative yaw torques, which become more negative with increasing sideways speed (Figure 14A). Moreover, for all tested robot configurations combined, these stabilizing negative yaw torques increase with the wing dihedral angle (Figure 14C). Thus, these results are in accordance with our yaw torque model and the hypothesis made based on CFD simulations on flying fruit flies.

For all configurations of the robot—symmetrical and asymmetrical, when the yaw command is equal to 0 the flapping wing flyer experiences a yaw torque induced by sideways velocity. To observe the stabilizing effect of this, the wind velocity has to be in a certain range. The wind speed of 0.5 m/s does not produce a strong enough torque that would consequently turn the robot and keep it in the position - facing the wind. Whereas at high speeds it is harder to the control robot in the air, which is why we limited our experiments to a maximum wind speed of 1.5 m/s. Finding the optimal shift of the center of mass and an active control scheme of the mass displacement mechanism would be of great value to mitigate the lateral wind gusts better and quicker.

**FIGURE 15:**
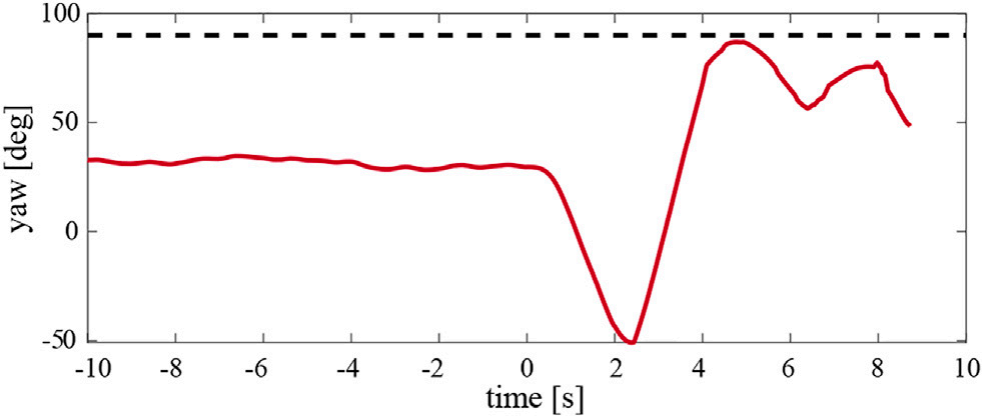
The temporal dynamics of the yaw angle of the hybrid case whereby the proportional term of the yaw control loop was reduced by 50%. This resulted in the stable hover and produced passive yaw torque. The robot had a 4 cm mass displacement configuration and encountered a side wind of 1.5 m/s. The 90° yaw angle, which corresponds to robot flying into the wind, is shown by the black dashed line.

The attitude control loop of the robot is based on a proportional-derivative controller. Therefore, deactivating the proportional term of the yaw axis during the experiments caused the flapping flyer to slowly drift during hover, which is not desirable for the real-life application of the discussed mechanism on a MAV. For this purpose, we present the final experiment where we consider a hybrid solution, shown in Figure 15. The P gain is reduced by 50% and as a result, the drift in the yaw axis is eliminated without affecting the passive torque generation mechanism. This suggests that the passive lateral gust mitigation strategy can be directly applied in flapping wing MAVs.

**FIGURE 16:**
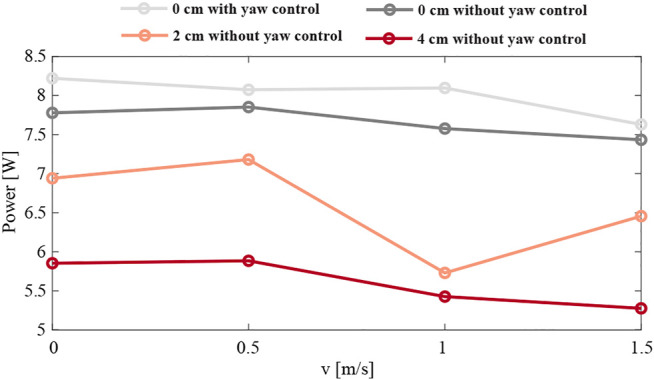
The average power consumption of the robot flying in a side-wind with various wind speeds, and in four different configurations: with a 0 cm mass displacement with and without yaw control (light and dark grey, respectively), and without yaw control and a mass displacement of 2 and 4 cm (orange and red, respectively). The average power consumption was determined during a 5 s time window in which the robot transitioned from sideways flight to forward flight in the wind gust.

Moreover, to indicate the usability of the asymmetric scheme, we investigate the power consumption during all the experiments. We collected power averages from a phase of the flight occurring after 5 s. Figure 16 shows the results. The highest power consumption of 8.2 W is observed for the symmetric configuration with full body attitude control at hover. Once the yaw control is turned off, the power consumption slightly decreases by 0.4 units. The most efficient is the 4 cm case, where the power usage drops below 6 W, which can be related to the positive dihedral angle, resulting in the wings providing extra lift. Moreover, with increasing wind speed, we can observe the inversely proportional changes in the power curve.

Finally, we remark that due to practical constraints, we report here on one experiment per condition. For example, we performed 16 experiments for the four wind speeds and four configurations and an additional test for the hybrid case where we eliminate the yaw drift. Although the main findings correspond to the expectations and to the results of preliminary, non-reported experiments, more knowledge on the precision and uncertainty bounds would require an even more extensive experimental campaign, which we consider as future work. We would like to also mention that we did not consider here influence of the actuators or the controller parameters on the system dynamic behaviour. We acknowledge that different conditions and settings may result in different responses. Moreover, counter-torques can also lead to over-compensation, and thus precise tuning of the flight platform design is still required. Although we did not observe such an effect in our robotic flyer, further investigation is needed to evaluate the risk of overturning, and how this depends on flight platform design.

### 4.3 Flapping Flyers Are Robust to Sideways Wind Perturbations

Flapping flyers with a positive stroke dihedral have a baseline stability to sideways wind gusts. To reject a sideways wind gust, a flapping flyer needs to only produce pitch down torque. This allows them to accelerate against the wind, and as this action increases stroke dihedral it simultaneously augments the aerodynamic yaw torque coupling with sideways air gusts. The indicated causes the flyer to turn into the wind, further enhancing the lateral gust rejection dynamics.

This shows that flapping flight systems are strikingly robust against wind perturbations, especially when they have a wingbeat with positive stroke dihedral. It might also explain why a large number of flying animals possess this stroke dihedral during their unperturbed flight. Interestingly, some highly-maneuverable insects such as hoverflies and dragonflies have a relatively small wingstroke dihedral angle. It might be that to boost their maneuverability, these animals have reduced gust-rejection capabilities, suggesting that there is a trade-off between maneuverability and gust-rejection capabilities in flapping flyers. In addition, there might be other strategies that animals use based on their abilities, predispositions, or in accordance with other existing environmental factors.

The robot experiments showed that the robot with close-to-zero dihedral angles is very susceptible to wind gusts in hovering conditions. By shifting the mass below the center of mass backward, we automatically introduced a positive stroke dihedral while hovering, after trimming. This is equivalent to the rearward-located abdomen mass in insects. The positive stroke dihedral greatly enhanced the passive stability of the robot in the presence of wind gusts. This shows that to make flapping robotic flyers more robust against wind gusts, one can take inspiration from nature by introducing a positive stroke dihedral using a dorsal-ventral mass asymmetry. By making this mass-shift system adjustable in flight, as we did here, one can trim from a stable system to a more maneuverable system depending on the current situation and requirements.

## Data Availability

The original contributions presented in the study are included in the article/Supplementary Material, further inquiries can be directed to the corresponding author.
