# Supplementary material for: Flying Into the Wind: Insects and Bio-Inspired Micro-Air-Vehicles With a Wing-Stroke Dihedral Steer Passively Into Wind-Gusts
Source: Front Robot AI. 2022 Feb 24;9:820363. doi: 10.3389/frobt.2022.820363 (PMC8907628; doi:10.3389/frobt.2022.820363)
Supplement: Supplementary file 5 [file DataSheet1.pdf]

Supplementary Material

1 SUPPLEMENTARY FILE LEGENDS

Dataset S1. Results of all CFD simulations with hovering and sideways flying fruit flies, in CSV-file format. Each row shows data for a single simulation, and includes the pitch torque mode, stroke dihedral angle (in degrees), sideways flight speed (m/s), and normalized aerodynamic torques and forces. See figure 1f for the definitions of the parameters.

Movie S1. Visualization of the airflow around a hovering fruit fly (V = 0 m/s) with the wingbeat pattern of Pitch Torque Mode = 0, as determined using a CFD simulation. In the video, the fly is shown from above, and the airflow is visualized using iso-surfaces of constant vorticity (4000 and 5000 s^{-1}), color-coded with the air-pressure coefficient at the surfaces. See figure 9 for the air-pressure coefficient color bar.

Movie S2. Visualization of the airflow around a hovering fruit fly (V = 0 m/s) with the wingbeat pattern of Pitch Torque Mode = 2, as determined using a CFD simulation. In the video, the fly is shown from above, and the airflow is visualized using iso-surfaces of constant vorticity (4000 and 5000 s^{-1}), color-coded with the air-pressure coefficient at the surfaces. See figure 9 for the air-pressure coefficient color bar. Stills of this video at the wing-stroke reversals are shown in figure 9A.

Movie S3. Visualization of the airflow around a hovering fruit fly (V = 0 m/s) with the wingbeat pattern of Pitch Torque Mode = -2, as determined using a CFD simulation. In the video, the fly is shown from above, and the airflow is visualized using iso-surfaces of constant vorticity (4000 and 5000 s^{-1}), color-coded with the air-pressure coefficient at the surfaces. See figure 9 for the air-pressure coefficient color bar. Stills of this video at the wing-stroke reversals are shown in figure 9B.

Movie S4. Visualization of the airflow around a sideways flying fruit fly (V = 1 m/s) with the wingbeat pattern of Pitch Torque Mode = 0, as determined using a CFD simulation. In the video, the fly is shown from above, and the airflow is visualized using iso-surfaces of constant vorticity (4000 and 5000 s^{-1}), color-coded with the air-pressure coefficient at the surfaces. See figure 9 for the air-pressure coefficient color bar.

Movie S5. Visualization of the airflow around a sideways flying fruit fly (V = 1 m/s) with the wingbeat pattern of Pitch Torque Mode = 2, as determined using a CFD simulation. In the video, the fly is shown from above, and the airflow is visualized using iso-surfaces of constant vorticity (4000 and 5000 s^{-1}), color-coded with the air-pressure coefficient at the surfaces. See figure 9 for the air-pressure coefficient color bar. Stills of this video at the wing-stroke reversals are shown in figure 9C.

Movie S6. Visualization of the airflow around a sideways flying fruit fly (V = 1 m/s) with the wingbeat pattern of Pitch Torque Mode = -2, as determined using a CFD simulation. In the video, the fly is shown from above, and the airflow is visualized using iso-surfaces of constant vorticity (4000 and 5000 s^{-1}), color-coded with the air-pressure coefficient at the surfaces. See figure 9 for the air-pressure coefficient color bar. Stills of this video at the wing-stroke reversals are shown in figure 9D.

Movie S7. Free flight experiment of the flapping-wing MAV with the low-speed multi-fan wind system. The robotic flier is in the symmetrical configuration - 0 cm mass displacement, flying at 1.5 m/s wind speed. The recording does not contain the starting phase which is reaction to step increase in the sideways wind. We can see that the MAV turns around and oscillates between its natural equilibrium - facing the wind.

Movie S8. Free flight experiment of the asymmetric flapping-wing MAV with the low-speed multi-fan wind system. The robotic flyer is in the configuration with 2 cm mass displacement and is flying at 1.5 m/s wind speed. The recording does not contain the starting phase which is the 90 degrees turn of the robot while encountering step increase in the sideways wind. Nevertheless, we can still observe that the flapping flier in this configuration produces a more consequent nose-down pitching moment. We can also see a tendency to perform diverging oscillations around its equilibrium state.

Movie S9. Free flight experiment of the asymmetric flapping-wing MAV with the low-speed multi-fan wind system. The robotic flyer is in the configuration with 4 cm mass displacement and is flying at 1.5 m/s wind speed. The recording does not contain the starting phase which is the 90 degrees turn of the robot while encountering step increase in the sideways wind. This configuration at best presents the stabilizing effect of the passively induced yaw torque. After a turn of around 90 degrees induced by side wind of 1 and 1.5 m/s, the robotic flyer continues to keep its body attitude. The yaw angle is converging to around 90 degrees, corresponding to forward flight in the incoming wind gust with a substantial pitch angle

Movie S10. Free flight experiment of the flapping-wing MAV in the attitude stabilization mode without yaw control. The pilot controls the roll angle via command sent from the transmitter. The interaction of the wings with sideways speed induces passive yaw torque.
